# Middle Lobe Syndrome Associated with Bronchial Anthracofibrosis

**DOI:** 10.15388/Amed.2020.27.2.8

**Published:** 2020-12-21

**Authors:** Ugur Gonlugur, Tanseli Gonlugur, Sule Ozer

**Affiliations:** Canakkale Onsekiz Mart University, School of Medicine, Department of Chest Diseases, 17100, Canakkale, Turkey, Orcid id: 0000-0001-8720-2788; Canakkale City Hospital, Department of Chest Diseases, 17100, Canakkale, Turkey Orcid id: 0000-0003-0751-6184; Canakkale Onsekiz Mart University, School of Medicine, Department of Radiology, 17100, Canakkale, Turkey Orcid id: 0000-0002-9296-9489

**Keywords:** Bronchial diseases, Pigmentation, Anthracosis

## Abstract

Bronchial anthracofibrosis has been defined as airway narrowing associated with dark pigmentation on bronchoscopy without an appropriate history of pneumoconiosis or smoking. We present a case of a 67-year-old, non-smoking female patient who was referred to our clinic for two years of persistent cough. Spirometry was within normal limits. Radiological evaluation showed right middle lobe atelectasis and positron emission tomography-positive mediastinal lymph nodes. Bronchoscopy revealed black airway discoloration and distortions. In conclusion, we propose monthly radiological controls before an invasive procedure in such cases if a strong suspicion of malignancy/tuberculosis is not present.

## Abstract (lt)

**Vidurinės skilties sindromas, siejamas su bronchine antrakofibroze:**

**Santrauka. **Bronchinė antrakofibrozė – kvėpavimo takų susiaurėjimas, kai, atlikus bronchoskopiją, matoma tamsi pigmentacija, o duomenų, kad pacientas būtų sirgęs pneumokonioze ar rūkytų, nėra. Pristatomas 67 m. nerūkančios pacientės, kuri buvo siunčiama į mūsų kliniką dėl dvejus metus išliekančio kosulio, atvejis. Spirometrijos tyrimo rezultatai atitinka normos ribas. Radiologinio tyrimo metu nustatyta dešiniosios vidurinės skilties atelektazė, o pozitronų emisijos tomografijos tyrimo rezultatas tarpuplaučio limfmazgiuose buvo teigiamas. Atlikus bronchoskopiją matomi pajuodę ir deformuoti kvėpavimo takai. Apibendrinant reikia pasakyti, kad tokiais atvejais, jei piktybinių darinių / tuberkuliozės neįtariama, prieš atliekant invazinę procedūrą, rekomenduojama kas mėnesį atlikti radiologinius tyrimus.

**Raktažodžiai:** bronchų ligos, pigmentacija, antrakozė

## Introduction

Endobronchial discoloration with luminal narrowing was first described in 1951 by AbrahamCohen when he reported middle lobe narrowing in eight female patients. Six of 8 patients had anthracotic pigmentation in the right middle lobe due to perforated tuberculous lymph nodes [1]. Chung coined the term “anthracofibrosis” in 1998. They reported 28 never-smokers with considerable history of wood smoke exposure in Korea [2]. If pigmentation occurs alone, without anatomical distortion of the bronchi, this entity is known as bronchial anthracosis. Both cigarette smoke and air pollution can cause anthracosis [3]. Anthracosis can also affect liver, spleen, esophagus, and sinuses [4].

Anthracotic pigmentations are generally considered as the result of carbon particle deposition but cadmium, lead, iron, phenol, silica, silicates (mica, kaolin), asbestos, hydrocarbon complexes, also can cause airway pigmentation [5,6]. The pigmentations can usually be observed in patients without environmental exposure to coal dust or smoking. If deposited mineral particles cause fibrosis of the bronchial wall or surrounding interstitium which may cause hypertrophy of the bronchial wall and luminal narrowing, this term is known as bronchial anthracofibrosis [6]. The aim of this paper is to describe clinical characteristics and imaging manifestations of the disease. 

## Case report

A 67-year-old woman presented with 2 years of productive cough and breathlessness. The patient was farmer, never smoked but had indoor cooking/heating smoke exposure history. She was treated with antibiotics and bronchodilators by her primary care physician but did not improve her symptoms. Lung auscultation and cardiac evaluation was unremarkable. Forced vital capacity (FVC), 2.42 L (108%); forced expiratory volume in 1 sec (FEV1), 1.69 L (92%); FEV1/FVC ratio 70% in spirometry. 

**Figure 1.**
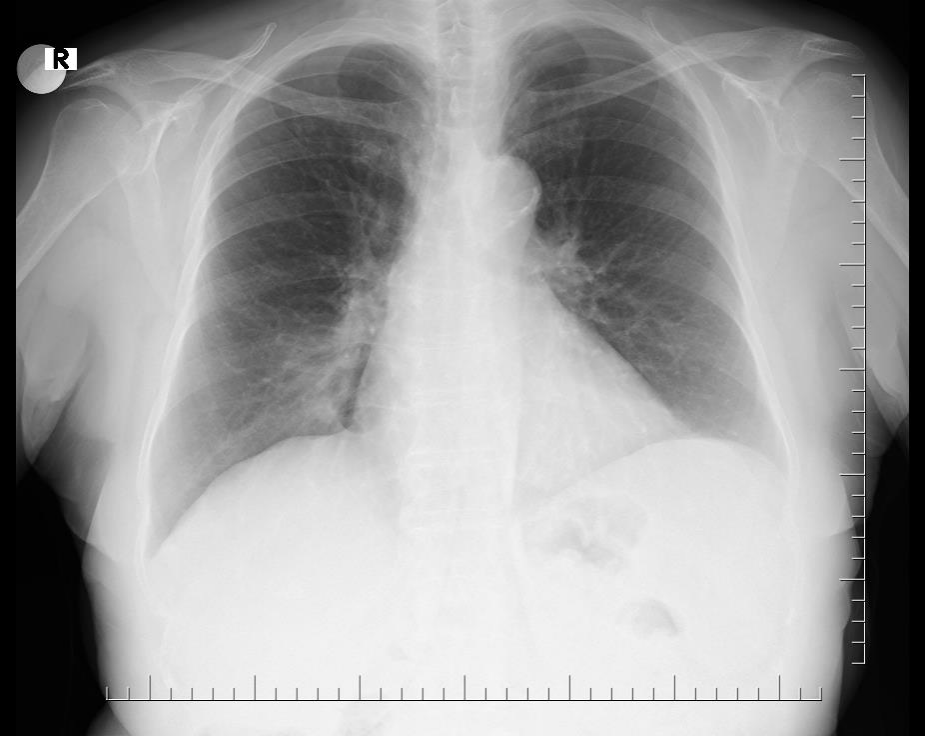
Chest X-ray showing right paracardiac ill-defined opacity.

**Figure 2.**
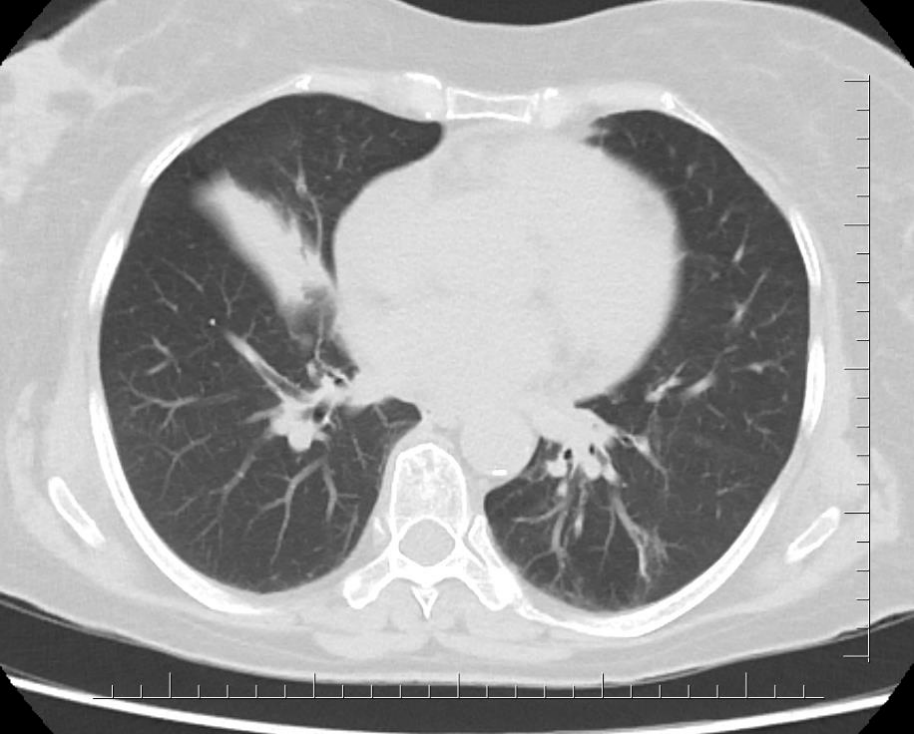
Chest CT-scan demonstrating lateral segmental collapse of the right middle lobe.

Chest radiograph revealed paracardiac opacity in the right lower zone (Figure 1). Chest CT-scan suggested middle lobe atelectasis (Figure 2). QuantiFERON-TB Gold test was positive. 

**Figure 3.**
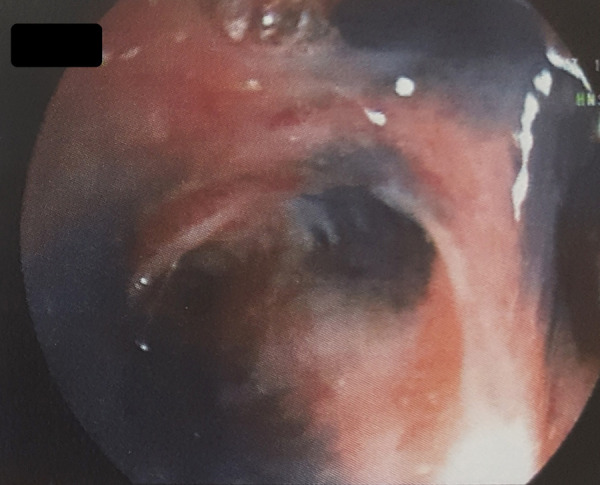
Black pigmentations and distortions on bronchial mucosa after the main bronchus.

There were metabolic activities in right hilar, subcarinal and right paratracheal mediastinal lymph nodes. Fiberoptic bronchoscopy demonstrated black pigmentation of the mucosa with narrowing and distortion of the right middle lobe without an endobronchial mass (Figure 3). Stains and cultures of the bronchial aspirate were negative for Mycobacterium tuberculosis. Biopsies from the mucosal discoloration confirmed anthracotic pigmentations and chronic inflammatory changes confirming diagnosis of bronchial anthracofibrosis. Both middle lobe atelectasis and positive me-diastinal lymph nodes on positron emission tomography were stable for two years. An informed consent has been obtained from the patient. 

## Discussion

Bronchial anthracofibrosis is observed among nonsmoking elderly women from developing countries who have exposure to biomass fuel smoke in poorly ventilated conditions. Most patients are often misdiagnosed with COPD without undergoing further workup to exclude bronchial anthra-cofibrosis [1]. 

Although bronchostenosis in anthracofibrosis tends to be multifocal, noncontiguous, and involves segmental or lobar bronchi in both lungs [7], it is more commonly (93%) occurred in right middle lobe bronchus [6,7]. Our patient had latent tuberculosis infection. If mineral accumulation exists in lymph nodes, tuberculosis cause rupture into the adjoining tracheobronchial tree, leading to black pigmentation, subsequent inflammation and fibrosis. Bronchial anthracofibrosis predominantly tends to affect middle lobe because of ineffective collateral ventilation. 

In the past, it has been suggested that tuberculosis is associated with bronchial anthracofibrosis. Regional lymph nodes were reactive because of chronic inflammation due to deposited mineral particles. The resulting tissue damage causes calcification. It has been reported that 85% of the cases showed lymph node enlargement involving hilar, peribronchial, and mediastinal nodes. The nodes were calcified in 91.7% of the cases, with 58% showing pressure effect on adjacent bronchi due to nodal enlargement [7]. Nodal enlargement due to particle depositions in lymph tissues increases as the biomass exposure period lengthens [6]. Confluence of enlarged lymph nodes can be seen both in anthracosis and malignancy, but nodal necrosis, which appeared in one third of the malignant nodes, is not observed in anthracosis [8]. Mediastinal lymph nodes can be PET negative [5] but bronchial anthracofibrosis can cause false positive PET-scan results for malignancy because flude-oxyglucose accumulates in inflammatory cells [8]. 

In conclusion, bronchoscopy is the gold standard in diagnosing anthracofibrosis. Because middle lobe atelectasis and false-positivity in PET can persist for long time, bronchoscopic biopsy should be undertaken in selective cases when a strong suspicion of malignancy/tuberculosis is present. Clinicians should be aware of bronchial anhracofibrosis in the differential diagnosis of calcified/enlarged hilar or mediastinal lymph nodes. 
